# Estimation of self-motion duration and distance in rodents

**DOI:** 10.1098/rsos.160118

**Published:** 2016-05-25

**Authors:** Magdalena Kautzky, Kay Thurley

**Affiliations:** 1 Department Biologie II, Ludwig-Maximilians-Universität, München, Germany; 2 Bernstein Center for Computational Neuroscience Munich, , Germany

**Keywords:** bisection task, interval timing, odometry, path integration, rodent navigation, virtual reality

## Abstract

Spatial orientation and navigation rely on information about landmarks and self-motion cues gained from multi-sensory sources. In this study, we focused on self-motion and examined the capability of rodents to extract and make use of information about own movement, i.e. path integration. Path integration has been investigated in depth in insects and humans. Demonstrations in rodents, however, mostly stem from experiments on heading direction; less is known about distance estimation. We introduce a novel behavioural paradigm that allows for probing temporal and spatial contributions to path integration. The paradigm is a bisection task comprising movement in a virtual reality environment in combination with either timing the duration ran or estimating the distance covered. We performed experiments with Mongolian gerbils and could show that the animals can keep track of time and distance during spatial navigation.

## 1. Introduction

Spatial cognition and navigational abilities require utilization of knowledge about one’s own location in relation to the surrounding environment. Such spatial knowledge is supposed to be formed from multi-sensory sources by integrating information about landmarks and self-motion over time and space [1]. Multi-sensory inputs provide the basis for the internal representation of space constructed in the hippocampal formation from place cells, head-direction cells, grid cells and other neurons with spatially selective properties [2]. The foundations of the hippocampal–entorhinal space representation have been thoroughly investigated in recent years and their possible role in computations necessary for navigation has been devised [3]. Related functional interaction has been reported with other cortical areas including visual and motor systems [4], parietal cortex [5,6], prefrontal cortex [7] and retrosplenial cortex [8]. However, less is known about how mammals sense the spatial characteristics of their environment. What do they extract from multi-sensory inputs to drive behaviour? In this study, we focus on self-motion cues. It is well established that insects make use of self-motion cues through visual odometry (e.g. bees, [9]) or proprioceptive odometry (e.g. ants, [10]). Also humans have been reported to be able to retrieve information from self-motion (e.g. [11–13]). Whether rodents possess similar capabilities has not been demonstrated so far. Studies on path integration in rodents typically only consider heading direction [11,14].

We designed a behavioural paradigm to probe time and distance estimation in rodents during self-motion. Our paradigm is a variant of the *bisection task* well known in interval timing research (e.g. [15,16]): in a two-alternative forced-choice experiment a subject has to categorize temporal intervals as ‘short’ or ‘long’ according to previously learned references. In our variant, we let Mongolian gerbils (*Meriones unguiculatus*) run along a hallway in virtual reality (VR) [17] for either a certain temporal interval or a virtual distance. Afterwards, the animals had to report whether the duration of the run or the distance covered was ‘short’ or ‘long’. Using VR, we could decorrelate running time and the simultaneously covered virtual distance, and hence specifically ask for one or the other. With our experiments, we demonstrate that gerbils are able to retrieve temporal and spatial information during self-motion. The behavioural paradigm may be adapted for other rodent species and may hence extend research on spatial navigation and its neural foundations.

## 2. Material and methods

### 2.1. Animals

Experiments were performed with seven adult male Mongolian gerbils (*Meriones unguiculatus*). All animals were at least three months of age at the beginning of the experiments. The animals weighed between 65 and 85 g and received a diet which kept them at about 85–95% of their free feeding weight.

### 2.2. Experimental apparatus

**Figure 1.**
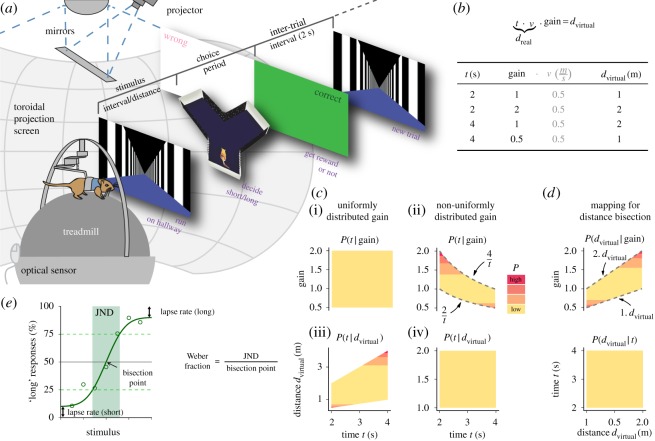
Design of temporal and spatial bisection experiments. (*a*) Experimental set-up and behavioural paradigm. For the experiments, a gerbil was placed into a VR set-up. The animal had to run along a virtual linear corridor and estimate either the duration or the visual/virtual distance covered. Afterwards, a virtual Y-shaped maze was presented, in which the animal had to categorize the stimulus into ‘short’ or ‘long’, compared with previously learned references, by running to the end of one of the two arms. For every correct choice, a food reward was delivered. In addition, visual feedback was given by setting the entire screen to either green (correct) or white (wrong) for 2 s. Finally, another trial was initiated. (*b*) During spatial behaviour running time *t* and distance *d*_*real*_/*d*_*virtual*_ are connected via the running speed *v*. To disentangle both, we changed the gain factor between treadmill movement and virtual movement. The table gives example values (see Material and methods for details). (*c*) Comparison of uniform ((i),(iii)) and non-uniform ((ii),(iv)) stimulus–gain mappings at the example of temporal bisection. Joint distributions are displayed for running time and gain factor ((i),(ii)), and running time and virtual distance ((iii),(iv)). The corresponding conditional probabilities of time given a particular gain *P*(*t*|gain) and of time given a particular virtual distance *P*(*t*|*d*_*virtual*_) are overlayed in colours (see colour bar). A uniform time–gain mapping introduces correlations between running time and covered virtual distance ((i),(iii)). Such correlations are avoided, when gains are chosen from the interval [2/*t*,4/*t*] (grey dashed lines in (ii)), although this introduces a negative correlation between time and gain. We used non-uniform mapping in our experiments. (*d*) For spatial bisection, we also used a non-uniform mapping. Gain values were chosen from the interval [1⋅*d*_virtual_,2⋅*d*_virtual_]. Note that here we have a positive correlation between distance and gain. (*e*) Illustration of psychometric parameters extracted from experimental data. The bisection point is defined as the stimulus which produces 50% ‘long’ responses and the just noticeable difference (JND) as the range of stimuli between 25 and 75% ‘long’ responses.

We used a VR set-up for rodents (figure 1*a*). For a detailed description, see [17]. In brief, the set-up comprises a styrofoam sphere that acts as a treadmill. On top of the sphere, an animal is fixated with a harness that leaves head and legs freely movable. Rotations of the sphere are induced when the animal moves its legs, but the animal itself stays in place. The rotations are detected by two infrared sensors connected to a computer that generates and updates a visual virtual scene. The scene is displayed via a projector onto a projection screen that surrounds the treadmill. For real-time rendering, we used Vizard Virtual Reality Toolkit (v. 5, WorldViz, http://www.worldviz.com). The virtual environments were designed with Blender (v. 2.49b, http://www.blender.org/).

### 2.3. Behavioural paradigm

We trained gerbils to either estimate the duration or the visual/virtual distance covered while running down a virtual linear hallway and then categorize this stimulus into ‘short’ or ‘long’ according to previously learned references (figure 1*a*). The hallway was of infinite length and its walls were textured with a repetitive pattern of black and white stripes, to exclude that the animals could use landmark-based strategies for task-solving. At the beginning of a trial, an animal faced the far end of the hallway. Time or distance measurement, respectively, was initiated when the animal started running. The animal had to continuously run with a speed of at least 0.1 m s^−1^, otherwise the trial started anew. Virtual position was sampled at 10–100 Hz. When target time or distance were reached, the hallway disappeared. For a brief period (0.5–2 s), a black screen was displayed. Especially during training, this procedure was necessary to allow the animal to stop running and get prepared for the choice period. Afterwards, the animal was ‘teleported’ into the stem of a virtual Y-shaped maze, implementing a two-alternative forced choice situation. Here the animal had to categorize the time or distance covered. For ‘long’ stimuli, the animal had to enter one of the two Y-arms, for short stimuli the other arm was correct. A timeout took place if the animal did not make a decision within 30 s. Following a correct choice, the animals received a food reward (Nutri-plus gel, Virbac, Bad Oldesloe, Germany or 20 mg pellets, TestDiet, St Louis, MO, USA). In addition, visual feedback was given by setting the entire projection screen either to green (correct) or white (wrong) for two seconds—two colours that gerbils can distinguish well [18]. Finally, the animal was reintroduced into the virtual linear maze to initiate another trial.

### 2.4. Disambiguation of time and space

During locomotion, time and distance are proportional to each other (formula in figure 1*b*) and both need to be un-confounded to test for one or the other. In our VR set-up, we achieved separation between running time *t* and virtual distance *d*_virtual_ by changing the coupling (gain factor; figure 1*b*) between treadmill and virtual environment. With a gain less than 1, running time *t* and distance covered on the treadmill *d*_real_ are larger than the virtual distance *d*_virtual_; and vice versa with a gain more than 1. The table in figure 1*b* illustrates how this may be used to un-confound running time *t* and virtual distance *d*_virtual_. The gain factor can be chosen such that different distances *d*_virtual_ are covered for the same duration *t* of movement. For instance, with gains of 1 and 2, one and two metres of distance are covered, respectively, during 2 s of movement at a speed of 0.5 m s^−1^. The same distances are covered when gains of 1 and 0.5 are combined with 4 s of running. Turning the picture around, we see that moving a virtual distance *d*_virtual_ of 1 or 2 m, may require 2 or 4 s of running, depending on the particular gain. Note that, by changing the gain, the duration *t* of the run and the distance covered on the treadmill *d*_real_ cannot be separated (cf. formula in figure 1*b*). Nevertheless, variability in an animal’s running speed will reduce the correlation between *t* and *d*_real_.

**Figure 2.**
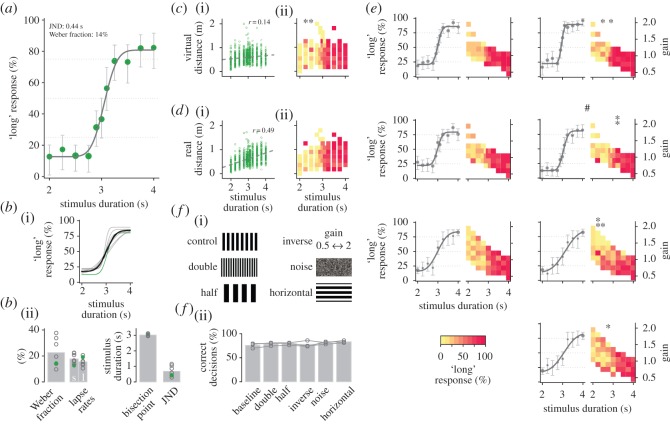
Temporal bisection. (*a*) Psychometric data for an example animal, i.e. percentage of responding ‘long’. Symbol size is proportional to the number of trials included in each data point (trial numbers were very similar in this example). Error bars are binomial confidence intervals. The thick solid line is the fit of a scaled cumulative Gaussian. (*b*) Psychometric data are similar for all animals. Psychometric curves (i) and parameters (ii) for all animals; grey lines or open circles, respectively; green line and circles indicate the animal from (*a*). Black line in (*b*(i)) depicts the average psychometric function. Bar graphs in (*b*(ii)) indicate averages. Individual lapse rates are given for short ‘s’ and long ‘l’ choices. (*c*) Responses do not depend on virtual distance. (i) Lack of correlation between stimulus duration and virtual distance for the animal in (*a*). Dashed line is a linear fit and *r* denotes Pearson’s coefficient of correlation. (ii) Subdividing the responses for different virtual distances displays no systematic effects. Percentage ‘long’ responses in each bin is colour-coded; cf. colour bar in (*e*). Asterisks mark significant differences in the responses within one column (*χ*^2^-test of homogeneity). (*d*) Responses do not depend on real distance. Stimulus duration and real distance are weakly correlated (i), but responses to the same stimulus do not display systematic dependence on real distance (ii). Illustration as in (*d*). (*e*) Responses do not depend on gain between treadmill and projection. Each pair of panels shows data for one animal. Left panels display psychometric data; illustration like in (*a*). Right panels depict response data sorted by gain. Illustration like in (*c*,*d*). Percentage ‘long’ responses in each bin is colour-coded; cf. colour bar. Panels corresponding to the animal in (*a*) are marked by #. (*f*) Manipulations to the virtual environment do not affect performance. (i) Illustration of the different manipulations. (ii) Percentage of correct decisions for manipulations comprising double and half period of vertical stripes, inverse gain distribution, random dot pattern (noise) and horizontal stripes. Grey open circles connected by lines mark data from the same animal (*n*=3). Bars display averages across animals.

**Figure 3.**
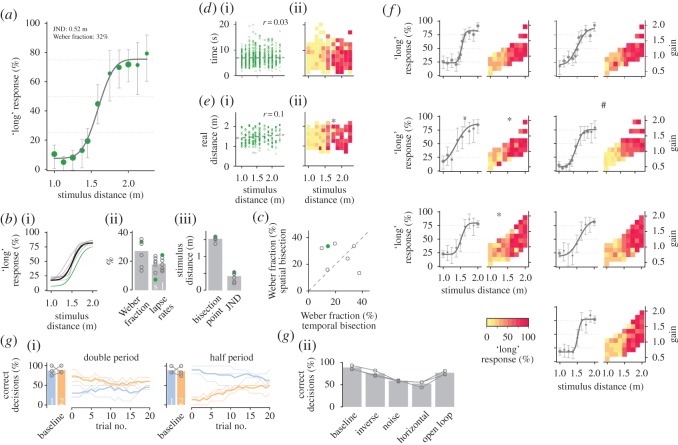
Spatial bisection. Illustrations in (*a*–*f*) same as in figure 2*a*–*f*. (*a*) Psychometric data for the example animal from figure 2*a*. Again symbol size is proportional to the number of trials included in each data point. (*b*) Psychometric curves (i) and parameters ((ii),(iii)) for all animals. (*c*) Correlation plot of the Weber fractions for temporal and spatial bisection for all animals (grey open circles; green circle indicates the animal from *a*). (*d*) Responses do not depend on running. (i) Lack of correlation between stimulus distance and running time for the animal in (*a*). (ii) Responses sorted for different running times display no systematic effects. (*e*) Responses do not depend on real distance. (*f*) Responses do not depend on gain. Illustration like in figure 2*e*. Again each pair of panels shows data for one animal. Data from the animal in (*a*) is marked by #. (*g*) Experiments with manipulations of the virtual environment. (i) Effects of doubling and halving the period of the vertical stripe pattern. In both cases, about 10 baseline trials were acquired before the experiment to ensure stable performance (bar graphs, averages for each reference; connected open circles, individual animals *n*=3). Then the manipulation was introduced. Here, decisions are plotted for each trial (running average of 10 trials). Thin lines represent individual animals, thick lines depict averages across animals. Blue indicates trials with 1 m and orange trials with 2 m. (ii) Responses for manipulations comprising inverse gain distribution, random dot pattern (noise), horizontal stripes and open-loop conditions. Grey open circles connected by lines mark data from individual animals (*n*=3). Bars display averages across animals.

Stimuli and gain factors used during training and control experiments (cf. figures 2*f* and 3*g*) were as listed in the table in figure 1*b*: in temporal bisection, gains of 1 and 2 were chosen for ‘short’ stimuli (2 s), and gains of 0.5 and 1 for ‘long’ stimuli (4 s). For spatial bisection, gains were 0.5 and 1 with ‘short’ (1 m), and 1 and 2 with ‘long’ stimuli (2 m).

For measuring psychometric curves in the test phase, also intermediate stimuli were presented. In addition, we picked gain factors from a range of values (cf. figure 1*c*,*d*): for temporal bisection, gains were sampled from the interval [2/*t*,4/*t*], i.e. the interval between short reference (2 s) divided by temporal stimulus *t* and long reference (4 s) divided by stimulus *t*. For spatial bisection, the range of gains was delimited by [1⋅*d*_virtual_,2⋅*d*_virtual_], i.e. values were chosen between short reference (1 m) multiplied by distance stimulus *d*_virtual_ and long reference (2 m) multiplied by stimulus. With these mappings, we end up with similar running times and virtual distances in both temporal and spatial bisection. Moreover, such an elaborate gain-mapping is necessary to fully disentangle both running time and virtual distance in a two-alternative forced choice setting. Figure 1*c* illustrates the situation for temporal bisection: with a uniform mapping between temporal stimulus and gain, the running time *t* still carries information about the virtual distance *d*_virtual_. Short running times may lead to short virtual distances and long running times to long distances. This correlation may be exploited by the animal for categorizing short and long stimuli. This can be avoided with non-uniform stimulus–gain mapping. However, then information is embedded in the gain, such that a subject may use the speed of virtual movement for task solving. To demonstrate that our animals did not respond to the gain but to the intended stimulus, we performed specific post hoc analyses detailed in Results.

### 2.5. Experimental schedule

Animals performed one session per day. Each experimental session lasted until the animal had made at least 20 trials, or, during training, until 30 min had passed. Trained animals typically performed between 30 and 40 trials per session; some animals achieved up to 60 trials.

Naive animals were accustomed to the VR set-up in a linear virtual maze for about 5–10 sessions (cf. [17]). Afterwards, we exposed the animals to the temporal bisection task. The animals were trained on two references, 2 and 4 s, for about 20 sessions. First, stimuli were not randomized but alternated for about 10 sessions. This period helped to familiarize the animals with the task and specifically the Y-shaped decision maze. All seven gerbils developed side preferences for one arm of the Y-maze. To counteract this behaviour, we interspersed training on the full task with sessions in which only the Y-maze was presented and the animal was only rewarded, when it chose the non-preferred arm. Typically, two to three such sessions were sufficient. In a second training step, the reference stimuli were randomized. After another 10 sessions, the animals reached decision levels well above chance and choice behaviour remained stable over sessions. Animals were trained until they were able to discriminate both references above chance for at least two sessions. During the test phase, intermediate stimuli were presented. Typically, the reference stimuli were over-represented to ensure stable performance levels. In some sessions, all stimulus values were presented equally often. The test phase took about 20 sessions for temporal bisection. With three of seven animals, we performed control experiments for five sessions after the test phase, in which we modified the virtual environment.

After finishing the temporal bisection experiments, we changed the task to spatial bisection. Relearning took about 15 training sessions in which we only presented two references, 1 and 2 m. We immediately started with randomized stimulus presentation. In the following test phase, we again presented intermediate stimuli. This phase lasted about 20 sessions. After the test phase, we again conducted control experiments with three of seven animals for six to seven sessions.

### 2.6. Analysis of behaviour

Data analysis was done with Python v. 2.7 using the packages Numpy v. 1.9, Scipy v. 0.16, Statsmodels 0.6 and Matplotlib 1.5. Psychometric functions were determined as the probability of choosing ‘long’. Confidence intervals for the responses were calculated as Clopper–Pearson intervals based on the beta distribution. We fitted the psychometric data and extracted several parameters (figure 1*e*) using a scaled variant of a cumulative Gaussian *F*(*x*)

2.1 $$\psi (x)=\lambda_{s}+(1-\lambda_{s}-\lambda_{l})F(x).$$

The variables λ_s∖l_ represent lapse rates for short and long responses, respectively. The point where *ψ*(*x*)=50% is defined as the bisection point, i.e. the stimulus at which 50% ‘long’ responses are produced. The range of stimuli *x* between *ψ*(*x*)=25 and 75% is the just noticeable difference (JND). The Weber fraction is given as JND divided by the bisection point. We used a Bayesian inference method to fit equation (2.1) to the data [19] implemented in Psignifit v. 3.0 [20]. Levels of significance are indicated in plots by *p<0.05, **p<0.01, ***p<0.001.

## 3. Results

We conducted two types of bisection experiments to investigate time and distance estimation during self-motion in Mongolian gerbils. All animals were first tested for bisection of temporal intervals and in a second series of experiments for bisection of virtual distances.

### 3.1. Temporal bisection

Figure 2*a* displays psychometric data of an example animal in the temporal bisection experiment. Plots for all animals are given in figure 2*e*. Datasets were acquired over 21 sessions and include more than 600 trials. The number of sessions and trials was similar for all animals. To quantify psychometric curves, we fitted cumulative Gaussians to the response data and extracted bisection points, JNDs, Weber fractions and lapse rates. The example animal in figure 2*a* was able to estimate temporal intervals with a precision of 0.43 s (JND; Weber fraction of 14%). Similar effects were found across all animals (*n*=7, JNDs 0.69±0.3 s, Weber fractions 23±10%; figure 2*b*). Bisection points were at 3.0±0.1 s and thus close to the arithmetic mean of the two references. Lapse rates lay between 10 and 20% and were slightly different between ‘short’ and ‘long’ responses, indicating weak preferences for one of the two arms of the Y-maze in individual animals.

To evaluate whether our approach to disentangling running time from virtual distance was successful, we reanalysed the data with respect to virtual distance. Stimulus duration and virtual distance were only weakly correlated for all seven animals (Pearson’s *r* 0.07±0.11, cf. figure 2*c* for the example animal). As a next step, we subdivided the responses to each stimulus duration for the virtual distances that were covered at the same time (figure 2*c* for the example animal and electronic supplementary material, figure S1a for all animals). No systematic effects became apparent that could be attributed to incomplete decorrelation. Furthermore, the animals did not modulate their running speed to account for differences in the optic flow introduced by the different gains between treadmill movement and virtual environment (Pearson’s *r* −0.08±0.08).

Stimulus durations were correlated with the real distance covered, i.e. distance on the treadmill (Pearson’s *r* 0.57±0.11 across all animals; figure 2*d*). Such a correlation is expected as our approach only permits uncoupling real, i.e. treadmill movement, from movement in the virtual environment. Real running distance and running duration remain correlated. Given this correlation, the question comes up whether animals responded to running time or real distance. However, this is hard to answer from psychometric plots. Therefore, we also subdivided the responses to each stimulus duration with respect to the real distance that was covered at the same time. No differences were present that could explain an animal’s responses (figure 2*d* and electronic supplementary material, figure S1b). This indicates that animals indeed responded to duration and not real distance.

By design, stimulus duration and gain factor were inversely correlated in the temporal bisection experiment (Pearson’s *r* −0.73±0.04; cf. figure 1*c*). We therefore examined if our results could have been due to using the gain, instead of the actual stimulus. Figure 2*e* displays the responses to each stimulus sorted by gain for each animal separately. Responses had similar probability across the respective gain factors for each stimulus duration. None of the panels in figure 2*e* looked similar to the theoretical prediction in figure 1*c*(ii), arguing against the possibility that animals exploited information from the gain for task solving.

For temporal bisection, features of the virtual maze, i.e. visual cues of self-motion, should not affect performance. To test this, we conducted experiments with three of our animals, in which we manipulated the maze. Each experiment included one modification, as illustrated in figure 2*f*(i). The animals performed one session with 40–60 trials for each modification. Only the two reference stimuli, 2 and 4 s, were presented. In the first two experiments, we doubled or halved, respectively, the period of the vertical stripe pattern covering the walls. Overall performance did not change and remained at baseline levels well above chance (Binomial test *p*<0.001 for each animal; figure 2*f*(ii)). In the next experiment, we choose the gains such that stimulus duration was proportional to virtual distance (i.e. not inversely proportional like in the normal experiments; cf. figure 1*b*,*c*). This ‘inverse’ gain-setting did not affect performance. Note that this also demonstrates that the gain was not used for responding by the animals. When we replaced vertical stripes with textures of random dots (noise, Julesz pattern) or horizontal stripes, performance was also similar to baseline conditions. A Friedman test indicated no differences in the performance across all modification experiments (*p*=0.2, *Q*_3,6_=7.2; figure 2*f*(ii)). Responses did not show strong changes throughout each experiment but were at similar levels (electronic supplementary material, figure S2).

### 3.2. Spatial bisection

In a second series of experiments, we switched to spatial bisection. This step is straightforward in our paradigm by asking for the virtual distance covered instead of the duration. The paradigm, including the virtual environment, remains unaltered in every other aspect. For relearning, we initially trained the animals with two reference stimuli. Training took about 15 sessions until the animals reached stable performance above chance. We aimed at matching the time range of the temporal bisection experiments and therefore chose 1 and 2 m as references and assumed an average running speed of 0.5 m s^−1^ (see also figure 1*b*). In the end, running times did not exactly match the range 2–4 s, since animals ran at speeds of 0.3±0.1 m s^−1^.

In the test phase, we again interspersed the range between the references with further stimulus values. Each animal made roughly 20 test sessions and 600 trials. Figure 3*a* displays psychometric data from the same animal as in figure 2*a*; data for all animals is provided in figure 3*f*. Psychometric parameters were at similar values for all seven animals (JNDs 0.41±0.13 m, Weber fractions 27±8%; figure 3*b*). Comparison of Weber fractions between temporal and spatial bisection did not reveal systematic effects across animals (figure 3*c*). Bisection points were at 1.5±0.1 m and thus again at the arithmetic mean between the two references. Lapse rates lay roughly between 10 and 20%. Note that the example animal in figure 3*a* displayed a strong preference for one arm of the Y-maze, which resulted in very different lapse rates for short and long choices, 7 and 24%, respectively. To test whether this behaviour remains beyond the ‘long’ reference, i.e. 2 m, we also presented stimulus distances of 2.125 and 2.25 m to this animal. The psychometric curve still saturated at the same response level.

As with temporal bisection, we evaluated if the animals could have made use of alternative strategies to solve the task. The stimulus, i.e. virtual distance, was not correlated with running time or real distance covered (Pearson’s *r* for virtual distance and time 0.05±0.05, and for virtual distance and real distance 0.09±0.1 across all seven animals; see figure 3*d*(i),*e*(i) for the example animal). Responses did not depend on time or real distance (figure 3*d*(ii),*e*(ii) and electronic supplementary material, figure S3). The animals did not modulate their running speed with the gain (Pearson’s *r* −0.06±0.03). Finally, we again determined whether the responses could be due to the gain instead of the actual stimulus distance, since both are correlated by design (Pearson’s *r* 0.69±0.08). However, responses to a particular stimulus were not modulated by the gain (figure 3*f*) and showed a different picture from the theoretical prediction in figure 1*d*.

For the virtual distance bisection task, animals had to extract movement information from the virtual environment. To probe their ‘visual odometer’ in more detail, we did a number of additional experiments with three of our animals. Each experiment comprised a different modification of the visual stimulation, similar to the temporal bisection experiments. We only presented the reference stimuli, 1 and 2 m. At the beginning of each experimental session, we did about 10 baseline trials with each animal to ensure stable performance. We typically tested a particular modification for only one session (around 40 trials). In the first two experiments, we doubled or halved the period of vertical stripes. If the animals were using the number of stripes as a means to estimate distance, we expected specific results, for both conditions: (i) When doubling the stripe period, the ‘long’ distance should still be recognized as long; the ‘short’ distance, however, would also fall into the ‘long’ category and correct responses should be below chance. Indeed, two of three animals displayed this choice behaviour for the first couple of trials (figure 3*g*(i) and electronic supplementary material, figure S4). After about 10 trials, the animals adapted their strategy. Note that we still rewarded correct responses to keep the animals motivated. (ii) For halved stripe periods, the reverse picture is expected: ‘short’ distances should still be categorized correctly but responses to ‘long’ distances should be below chance. This picture was present for the first couple of trials in all three animals (figure 3*g*(i) and electronic supplementary material, figure S2). After about 10 trials, however, animals again adapted their choice behaviour and responses converged to chance level.

Other modifications to the visual stimulation comprised (i) inverted gain, in which gains were chosen such that short (long) virtual stimulus distances resulted in short (long) running times (i.e. inverse setting compared to the normal experiments; cf. figure 1*b*), (ii) random dot texture (noise pattern), (iii) horizontal stripes, and (iv) open-loop experiments, in which runs along the hallway were independent of an animal’s movement. The inverted gain experiment did not affect the performance substantially and decisions were significantly above chance for all three animals (Binomial test *p*<0.01 for each of the three animals; figure 3*g*(ii)). Responses were similar throughout the whole experiment, even for initial trials, which otherwise could indicate rapid relearning (electronic supplementary material, figure S4). Similar to the results for temporal bisection, this demonstrates that animals did not base their decisions on the gain. When we covered the hallway’s walls with random dot textures or horizontal stripes, performance dropped to chance level for all three animals (figure 3*g*(ii)). Visual distance bisection is impossible with horizontal stripes, which do not provide any self-motion cue. Surprisingly, one animal correctly reported ‘short’ distances for the first couple of trials but responded below chance for ‘long’ distances—which can only be a random coincidence (electronic supplementary material, figure S4). However, visual distance bisection should be possible with random dot textures; although, extracting the relevant information may be hard. Here as well, two of three animals correctly reported ‘short’ distances initially but responded below chance for ‘long’ distances. Also, one of the two animals responded above chance for ‘long’ distances at later trials, which may be an indication that the animal learned to make use of the noise pattern for virtual distance estimation. However, further testing would be required to investigate this thoroughly.

In order to test whether the estimation of virtual distance is independent of the animal’s own movement, we performed experiments in an open-loop condition. As before, trials were initiated when an animal started to run, but then movement along the virtual track was played and hence decoupled from the animal’s own movement. Meanwhile animals usually kept running although they could also stop without any effect on the forward feed within the virtual maze. In the Y-maze, animals again had to move by themselves for responding. Correct decisions were significantly above chance for all three animals (Binomial test *p*<0.001, open loop in figure 3*g*(ii) and electronic supplementary material, figure S4). This experiment demonstrated that the animals indeed extracted all the information necessary to make their decisions from visual stimulation in the spatial bisection experiment.

## 4. Discussion

In this work, we investigated whether rodents are able to make judgements about temporal and spatial conditions of their own movement. We designed a novel bisection task that allowed for investigating temporal and spatial bisection separately but under identical conditions by decorrelating running time from running distance. The only change between different experiments was the specific task, i.e. timing the duration of running or judging the distance covered. As both running duration and running distance are inseparable under natural conditions, we implemented the task in VR [17,21,22], which allowed for a convenient solution to this problem. By changing the gain factor between an animal’s self-motion and the speed of movement within the virtual environment, we could decorrelate running time from virtual running distance. We demonstrated that Mongolian gerbils (*Meriones unguiculatus*) are capable (i) of keeping track of the duration of own movement and (ii) of using self-motion cues to estimate travel distance. Both capacities are instrumental to path integration and thus navigational abilities [3,11]. The task may readily be used with other rodent species.

### 4.1. Temporal bisection

The bisection task has been widely used to study temporal discrimination since its introduction by Church & Deluty [15]. For instance, it was applied in experiments with rodents, non-human and human primates (see [16], for a detailed overview). Being a two-alternative forced-choice experiment, the bisection task is useful to measure sensory thresholds (JNDs and Weber fractions).

Our results are in general consistent with reports from interval timing studies. Weber fractions in our temporal bisection task were close to what has been reported by others for sub- and suprasecond timing in rodents, i.e. about 20% (e.g. [15,23]). Bisection points were often reported to be sub-arithmetic or even at the geometric mean of the reference stimuli [16]. In our experiments, bisection points were close to the arithmetic mean for both duration and distance. We admit, however, that measuring bisection points may have been obscured in our experiments by biases due to side preferences of some animals in the Y-shaped decision maze. Such biases may lead to slight shifts of the psychometric curve along the abscissa.

### 4.2. Spatial bisection

Insects like bees are well known to use self-motion cues extracted from optic flow (visual odometry) for estimating travel distance [9,24,25]. Other insects, e.g. ants, use proprioceptive information (step-counter or pedometer) for path integration [10,26]. In mammals, evidence for visual odometry stems from studies in humans (e.g. [4,7,11–13,27,28]). Path integration experiments in rodents mostly focus on its directional component [11,14]. Descriptions of path integration for translational movements are rare. For instance, homing experiments with hamsters, showed their capacity in estimating distances from non-visual self-motion cues [29]. Other work on distance estimation in rodents used gap-jumping tasks [30,31], which are conceptually different from ours, since there distance estimation is not coupled to running. In some studies, the effect of visual stimuli in time-to-collision estimation was investigated with gerbils [32,33]: a visual target, whose size could be adapted dynamically, was presented at the end of a track. The animals had to run towards this target until contact; deceleration was used as a read out. When the target’s size was actively contracted (expanded) during the run, animals less (more) strongly decelerated. Such experiments demonstrated that rodents are able to make use of visual self-motion cues. We provide further evidence and extend previous work by demonstrating that rodents are able to visually discriminate path lengths. Moreover, we measured related discrimination abilities from psychometric functions. Visual odometry may thus be a strategy used by rodents for estimating distances covered during own movement.

Nevertheless, it remains open what kind of visual information the animals extracted in the spatial bisection experiment. Did they make use of optic flow or did they estimate the number of vertical stripes on the walls of the virtual environment they ran past? When we changed the stripe period, the animals made decision errors which would be predicted from a counting strategy. Also when we presented random dot textures, performance dropped, suggesting that it was the stripe-count that was used by the animals. However, it may be harder to infer distance from random dot patterns compared with stripes and additional training may be necessary [25]. Our results thus indicate that the animals measured distance by stripe-number estimation in our experiments. Further experiments are required to answer the question if rodents could also use optic flow information. Either way it is an interesting result that rodents are able to use visual self-motion cues for distance judgements—be it from stripe-number estimation or optic flow.

### 4.3. Problems of the virtual bisection task

By changing the gain factor between an animal’s running speed and the speed of movement within the virtual environment, we decorrelated running time from virtual running distance. Nevertheless, we could not fully eliminate some alternative strategies for task-solving in our experiments. The stimulus–gain mapping necessary for the two-alternative choice setting introduced correlations between stimulus and gain. Animals could thus have used the virtual speed itself for task-solving. Post hoc analyses, however, revealed that such a strategy was not used by our animals.

In the temporal bisection task, we could not exclude that animals were responding to the real distance covered on the treadmill, e.g. by counting steps. However, our analysis showed that usage of real distances for task-solving would less well explain the results. To avoid this problem, one could use a motorized treadmill and impose running speeds and hence real (foot-step) distance. This may reduce the amount of training, since the actual stimulus would become more apparent. Furthermore, with a means of imposing running speeds, our task could also be used to probe proprioceptive odometry (pedometry).

### 4.4. Potentials of the virtual bisection task: conclusion

The behavioural paradigm presented here may help investigating the foundations of path integration, when combined with recordings of neuronal activity. Neurons sensitive to temporal intervals have been shown, for instance, in parietal cortex [34], prefrontal cortex [35,36], hippocampus [37,38] and basal ganglia [39,40]. Hippocampal time cells are influenced by both running duration and running distance on a treadmill [41]. Furthermore, place cells may be involved in distance encoding [42,43], because their firing activity depends on visual, vestibular and proprioceptive information [44–46]. Optic flow input to the hippocampus has been found [47] as well as analogous activity patterns of neurons in visual cortex and place cells in hippocampus during spatial navigation [48]. The activity of neurons in visual cortex is modulated by optic flow [49,50].

Our novel behavioural paradigm extends the use spectrum of VR for rodents in particular with regard to investigations that are concerned with spatial navigation and its neural foundations [17,21,51,52]; in particular, if combined with recordings of brain activity—whether done with classical methods like tetrodes [43,46] or modern techniques such as calcium imaging of neuronal populations [52,53] and patch-clamp recordings from single neurons [51,54].

## Supplementary Material

Supplementary material

## Data accessibility

Datasets for all experiments reported in this paper are publicly accessible in the Dryad data repository at http://dx.doi.org/10.5061/dryad.4jd39.
