# Supplementary material for: Estimation of self-motion duration and distance in rodents
Source: R Soc Open Sci. 2016 May 25;3(5):160118. doi: 10.1098/rsos.160118 (PMC4892454; doi:10.1098/rsos.160118)
Supplement: Supplementary material [file rsos160118supp1.pdf]

Supplementary material

Estimation of self-motion duration and distance in rodents

Magdalena Kautzky and Kay Thurley

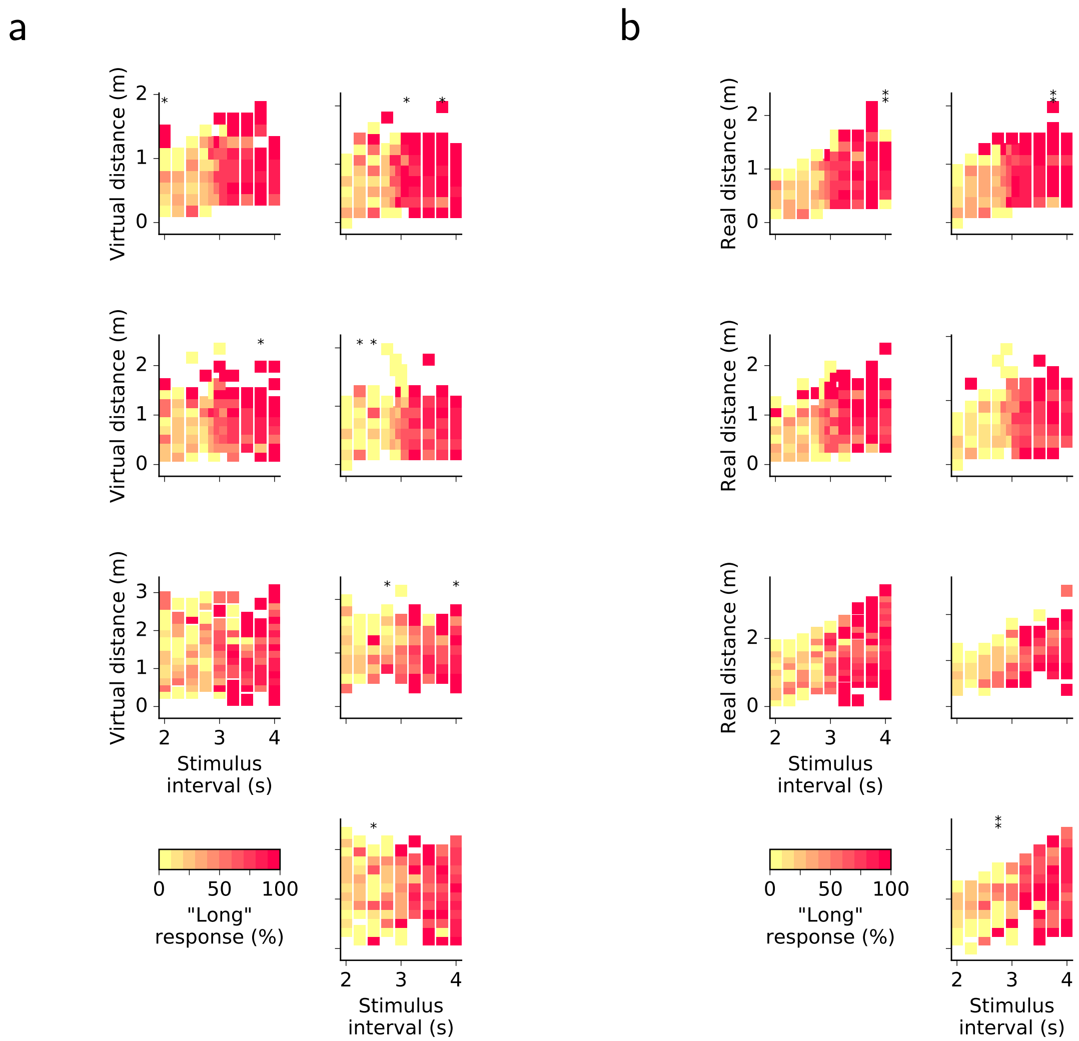

Figure S1. Relation of time and distance for temporal bisection. Responses to each stimulus interval are subdivided for virtual (a) and real distance (b). Each panel displays data from one animal. The arrangement of the panels is the same as in Figs. 2e&3f in the main text. Percentage "long" responses in each bin is color-coded; cf. colorbars. Asterisks mark significant differences in the responses corresponding to a particular stimulus according to a χ^2-test of homogeneity.

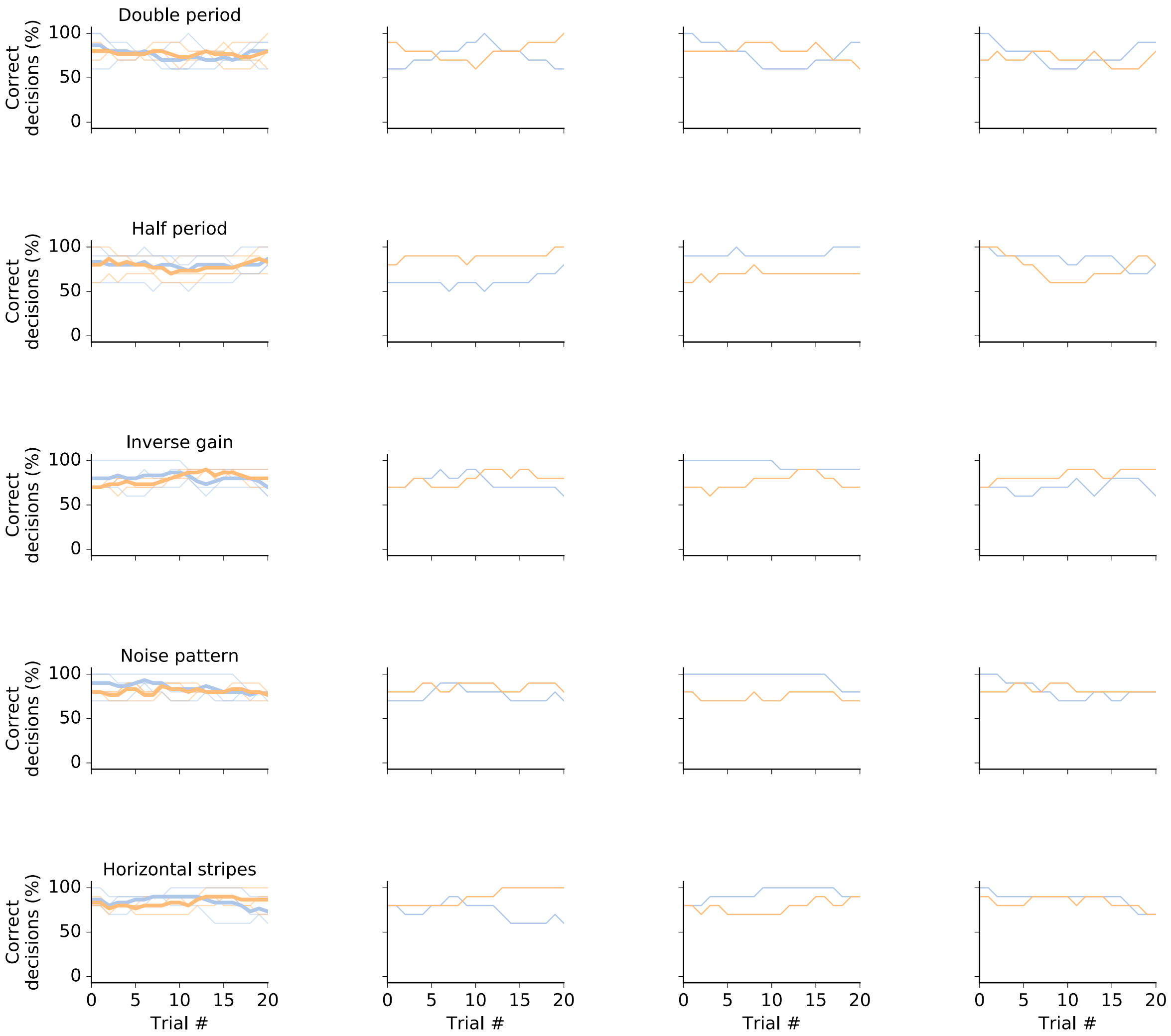

Figure S2. Temporal bisection with manipulations of the virtual environment. Manipulations comprised double and half period of the stripe pattern, inverse gain distribution, random dot pattern (noise), and horizontal stripes. Each row depicts data from one manipulation experiment. Decisions are plotted for each trial (running average of 10 trials). Thin lines represent individual animals, thick lines in leftmost panels depict averages across animals. Blue indicates trials with 2 s and orange trials with 4 s. For easier comparison the data for each animal is replotted in an individual panel in each row. Same order of panels for every manipulation.

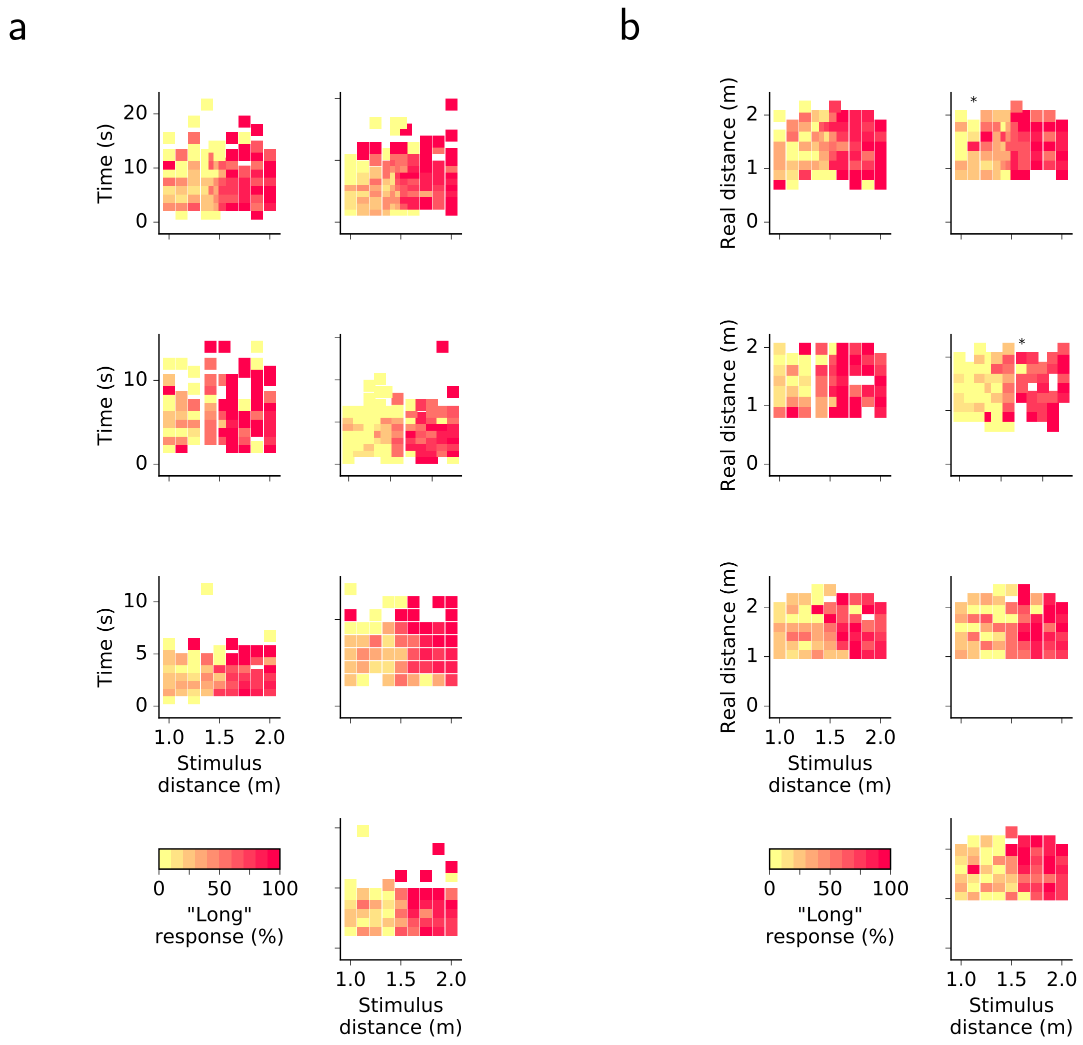

Figure S3. Relation of time and distance for spatial bisection. Responses to each stimulus distance are subdivided for running time (a) and real distance (b). Each panel displays data from one animal. The arrangement of the panels is the same as in Figs. 2e&3f in the main text. Percentage "long" responses in each bin is color-coded; cf. colorbars. Asterisks mark significant differences in the responses corresponding to a particular stimulus according to a χ^2-test of homogeneity.

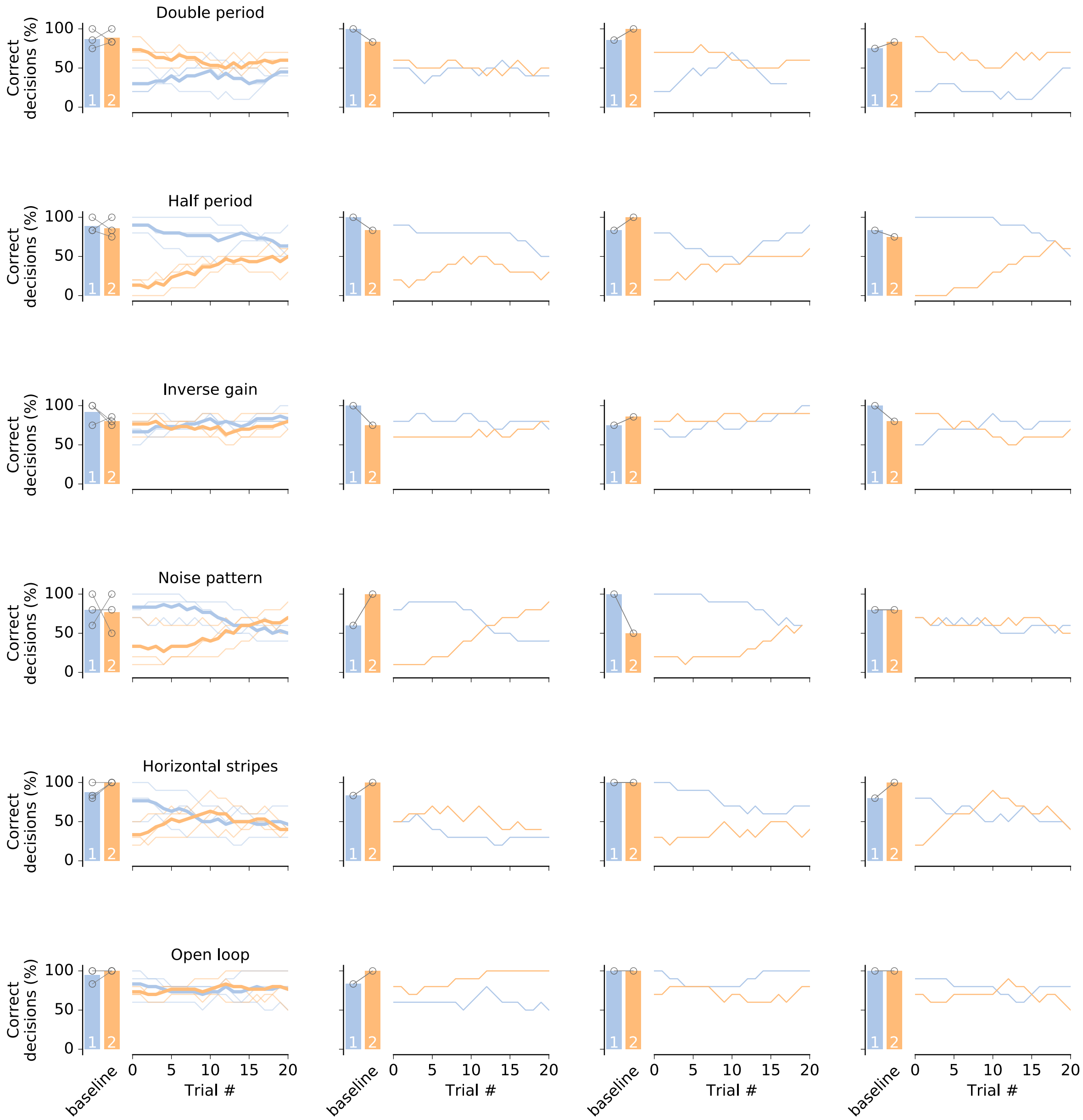

Figure S4. Spatial bisection with manipulations of the virtual environment. Manipulations comprised double and half period of the stripe pattern, inverse gain distribution, random dot pattern (noise), horizontal stripes, and open loop conditions. Each row depicts data from one manipulation experiment. About 10 baseline trials were acquired before the respective experiment to ensure stable performance (bar graphs, averages for each reference; connected open circles mark individual animals $n = 3$). Then the manipulation was introduced. Here, decisions are plotted for each trial (running average of 10 trials). Thin lines represent individual animals, thick lines in leftmost panels depict averages across animals. Blue indicates trials with 1 m and orange trials with 2 m. For easier comparison the data for each animal is replotted in an individual panel in each row. Same order of panels for every manipulation.
